# Exploring the Needs and Expectations of Inpatients Towards Assistive Technologies During Neuromotor Rehabilitation

**DOI:** 10.3390/healthcare14101355

**Published:** 2026-05-15

**Authors:** Giovanni Iaselli, Federico Colelli Riano, Pasquale Moretta, Simona Spaccavento, Laura Marcuccio, Ernesto Losavio, Gaetano Pagano, Francesco Amato, Giovanni D’Addio

**Affiliations:** 1 Istituti Clinici Scientifici Maugeri IRCCS, Laboratory of Bioengineering, Institute of Telese Terme, 82037 Telese Terme, Italy; giovanni.iaselli@icsmaugeri.it (G.I.); federico.colelliriano@icsmaugeri.it (F.C.R.); gianni.daddio@icsmaugeri.it (G.D.); 2 Istituti Clinici Scientifici Maugeri IRCCS, Department of Neuromotor Rehabilitation, Institute of Telese Terme, 82037 Telese Terme, Italy; laura.marcuccio@icsmaugeri.it; 3 Istituti Clinici Scientifici Maugeri IRCCS, Department of Neurorehabilitation, Institute of Bari, 70124 Bari, Italy; simona.spaccavento@icsmaugeri.it; 4 Istituti Clinici Scientifici Maugeri IRCCS, Neurology Unit, Institute of Bari, 70124 Bari, Italy; ernesto.losavio@icsmaugerri.it; 5 Istituti Clinici Scientifici Maugeri IRCCS, Laboratory of Bioengineering, Institute of Bari, 70124 Bari, Italy; geatano.pagano@icsmaugeri.it; 6 Department of Electrical Engineering and Information Technology, University of Naples “Federico II”, 80125 Naples, Italy; francesco.amato@unina.it

**Keywords:** rehabilitation, neuromotor disorder, assistive technology, stakeholder engagement, bioengineering

## Abstract

**Background:** technology can significantly improve quality of life for individuals with motor and cognitive disabilities, especially during the transition from rehabilitation to home. However, the success of assistive solutions depends on understanding patients’ functional status, needs, and attitudes toward technology. **Methods:** we investigated these factors using a multimodal assessment protocol, including the USASS questionnaire and ADL/IADL scales, among 69 participants admitted to intensive neuromotor rehabilitation, most of whom had caregivers (83.6%). Participants were grouped into three sample-derived ordinal categories (Low, Medium, High) based on ADL/IADL scores and age. **Results:** functional autonomy strongly predicts technology adoption and perceived benefits, surpassing age as a determinant. Individuals in the group with higher sample-derived functional levels reported greater independence in technology use and higher Quality Tech Impact scores. Correlation analysis confirmed moderate-to-strong positive associations between ADL/IADL scores and Quality of Life Index, with the strongest association observed for IADL (ρ = 0.703, 95% CI: 0.559–0.806, *p* < 0.001). These findings suggest that interventions should prioritise improving functional skills rather than focusing solely on age-related factors to enhance technology adoption and its positive impact. A proactive attitude toward technology is essential to avoid ineffective investments in assistive solutions. Ultimately, functional autonomy emerges as an important contributor of both technological engagement and overall well-being.

## 1. Introduction

In contemporary society, technology has become an integral part of daily life, supporting a wide range of professional activities where the adoption has a positive impact on output and productivity [1]. Beyond professional settings, technology also plays a crucial role in home-based healthcare, where patients continue recovery or manage long-term conditions after hospital discharge. In these contexts, digital platforms and smart devices enable remote monitoring and data exchange between patients, caregivers, and healthcare professionals [2,3]. Such connectivity enhances communication, promotes timely interventions, and reduces the need for in-person consultations [4].

The growing integration of telecommunication technologies in domestic healthcare reflects a paradigm shift towards proactive, patient-centred models of care that leverage continuous monitoring and data-driven decision-making [5]. In this regard, continuity of care has become increasingly important, making it essential to equip all stakeholders with appropriate digital tools to support telemedicine services [6]. This includes the use of specialised applications, services, and technologies within the home environment. A key development in this area is the integration of assistive technologies with home automation systems, which can significantly improve the quality of life and independence of individuals with disabilities or chronic conditions [7].

Before implementing such systems, however, it is crucial to assess the digital maturity of users, particularly patients. In healthcare, digital maturity has been described not only in terms of the availability and integration of digital systems, but also in relation to the capacity to use them effectively and generate meaningful benefits for care delivery and patient experience [8]. At the individual level, this concept also reflects people’s readiness, confidence, and ability to engage with digital tools in a meaningful way [9,10]. In the context of home-based neuromotor rehabilitation, digital maturity can therefore be operationally understood as the degree to which patients are prepared to interact with digital and assistive solutions, including their familiarity with technological tools, confidence in using them, and functional capacity to engage with smartphones, applications, sensors, and home automation platforms [10,11]. In the present study, the term assistive technologies was used in a broad home-rehabilitation sense and referred to digital and assistive solutions potentially supporting post-discharge from rehabilitation hospital setting, including smartphones and tablet-based applications, telemonitoring or sensing devices, and home automation or smart-home functions, rather than to a single specific device or product. Such preliminary assessments are essential to ensure that technological solutions are tailored to users’ specific needs and abilities. With the expected rise in the number of people with disabilities, the demand for assistive technologies and software designed to meet their unique requirements is growing rapidly. Ensuring the reliability of these tools requires building trust in user–device interactions and adhering to design principles that prioritise personalisation and effective implementation of Internet of Medical Things (IoMT) solutions in home care [12].

Within this framework, the Smart Ambient Assisted Living Ubiquitous System (SAALUS) research project serves as a reference initiative aimed at developing intelligent, adaptive environments through interconnectivity based on Internet of Things (IoT). By leveraging cloud technologies and smart sensors, the project seeks to enhance the quality of life of patients, support caregivers, and enable healthcare professionals to coordinate care more effectively [13].

Assessing digital maturity and technological proficiency across all stakeholders is therefore essential to ensure that innovation leads to practical, user-centred solutions in home-based healthcare. This topic has gained prominence in various fields, including healthcare management, where digital maturity assessment can optimise strategies, reduce inefficiencies, and improve the overall user experience [14].

Although previous studies have explored technology use, assistive technology uptake, and digital adoption in older adults or in people with disabilities, these investigations have often focused on community-dwelling populations, specific technology domains, or broader aging-in-place and smart-home contexts rather than on inpatients undergoing neuromotor rehabilitation and approaching discharge [15,16,17,18]. Moreover, the literature consistently highlights the importance of independence, usability, personalisation, and user needs in assistive technology provision, but comparatively, less attention has been devoted to how functional autonomy, digitally relevant attitudes, and expectations toward assistive solutions interact during the transition from inpatient rehabilitation to home-based care [19,20].

Questionnaire-based tools and maturity models are extensively utilised for the purpose of evaluating digital readiness and maturity across various levels, including patient, workforce, and organisational levels. These tools serve as a solid base for high-level planning and training initiatives. Nevertheless, a considerable gap persists within the extant evaluations, with assessments seldom contributing directly to the design of concrete telemedicine and home care technology solutions, particularly in relation to diverse and vulnerable demographics and features that are communication and engagement driven [21].

Within this context, this study aimed to explore, in a rehabilitation setting, how functional autonomy, age, and digitally relevant attitudes are associated with patients’ readiness and expectations toward assistive technologies for post-discharge home use.

In this regard, the primary objective of this study was to evaluate patients’ predisposition and ability to adopt technological tools designed to improve quality of life after discharge in relation to their clinical and functional characteristics, demographic variables, and digital maturity. More specifically, the study aimed to investigate how functional autonomy and digitally relevant attitudes shape expectations, perceived usefulness, and readiness to engage with assistive technologies in the transition from inpatient neuromotor rehabilitation to home-based care.

By focusing on this transition phase, the study contributes to the literature by linking functional status, patient expectations, and adoption-related attitudes within an applied home-care perspective, thereby informing the design of personalised assistive and smart-home solutions [22,23,24].

The initial section of this paper delineates the methodology employed in the design of the questionnaire structure and its clinical content. This methodology is supported by the introductory material in order to establish a direct link and justify the choices made. The third part of the study consists of the evaluation of the statistical analysis, the aim of which is to mathematically highlight the significance of the results obtained. These results serve as a starting point for the conclusions and discussion. These sections constitute the final part of the work, serving to reinforce the points made in the introduction through a critical approach. The objective is to establish a clear and evident correlation between the concepts introduced and those demonstrated.

## 2. Materials and Methods

We enrolled a consecutive series of patients admitted to Neurorehabilitation Units of Istituti Clinici Scientifici Maugeri IRCCS of Bari, according to following inclusion and exclusion criteria. Inclusion criteria included were as follows: (i) presence of neuromotor disease, (ii) age ranging between 18 and 80, and (iii) informed consent.

Exclusion criteria included the following: (i) presence of psychiatric or neurodegenerative disease; (ii) deficit in language comprehension. We collected personal relevant information potentially influencing digital attitude such as age, level of education, marital status, employment status, presence of caregivers or family members, onset date of illness, duration of hospital stays, and clinical diagnosis.

All patients were evaluated at the end of rehabilitation program, within one week before discharge from neuromotor rehabilitation units and before their return to home. All participants provided informed consent. The experimental procedures involving human participants described in this paper were approved by the Institutional Review Board of the local ethics committee “IRCCS Giovanni Paolo II, Bari.”

### 2.1. Assessment of Functional Independence, Digital Maturity, and Satisfaction

The evaluation of clinical features, attitude, and expectations toward the use of technology in their home-setting after discharge was performed by means of a multimodal battery including several scales. In detail, to assess the functional independence level of patients, we used two widely adopted scales in rehabilitation context such as (i) activities of daily living (ADL) and (ii) instrumental activities of daily living (IADL) [25]. To determine the digital maturity level of patients [26,27], we developed an ad hoc questionnaire called the Usability Assessment Questionnaire (USASS). The USASS is a structured instrument composed of 13 items designed to assess individuals’ familiarity with technology, attitudes toward assistive technologies, perceived benefits, and self-reported levels of autonomy and quality of life. The first section (Items 1–10) consists of ordinal, closed-ended questions rated on a 4-point Likert-type scale investigating prior experience with technology, enjoyment in its use, level of independence, availability of family support, and perceived impact of assistive technologies on quality of life, autonomy, and self-esteem as well as the importance of a target activity and satisfaction with its current performance. Items 1–7 and 9–10 are scored from 0 to 3, with higher scores indicating more positive attitudes, greater independence, and higher perceived benefit (0 = least positive response; 3 = most positive response). Item 8 is reverse-oriented and scored from 1 to 4, with higher scores indicating lower reliance on caregiver support. A composite score can be calculated by summing Items 1–10, yielding a total score ranging from 1 to 31, where higher scores reflect greater likelihood of technology adoption, higher perceived usefulness of assistive technologies, and greater independence. The second section (Items 11–13) includes three numeric rating scales ranging from 0 to 100 that assess perceived overall quality of life, autonomy, and quality of social relationships; these items are analyzed independently, with higher scores indicating better perceived outcomes. Overall, higher scores across the questionnaire indicate more favorable attitudes toward technology use and greater perceived well-being. The questionnaire showed high internal consistency with Cronbach’s alpha = 0.92. The adopted questionnaires are reported in Supplementary Materials.

### 2.2. Statistical Analysis

Data were stratified according to ADL scores, IADL scores, and age using a sample-derived distribution-based grouping strategy based on empirical quantiles. Quantile-based cut-points are commonly used to create ordered analytical strata for descriptive and exploratory comparisons when no externally validated clinical thresholds are available for the specific research question [28]. In addition, similar quartile-threshold approaches have been applied in exploratory studies to classify participants into ordered Low, Medium, and High groups [29]. For each variable, three ordinal categories were defined using the first quartile and the median as cut-points: values ≤ Q1 were classified as Low, values > Q1 and ≤Q2 as Medium, and values > Q2 as High. Accordingly, the High category included all observations above the median and did not correspond to a single quartile. These categories were therefore used as sample-derived analytical strata rather than clinically validated severity levels. Because categorising continuous or ordinal variables may reduce information compared with modelling them continuously [30], the present approach was restricted to exploratory non-parametric comparisons aimed at facilitating the comparison of response profiles across ordered bands of increasing functional autonomy or age. Modelling ADL, IADL, and age as continuous predictors was deemed to be beyond the scope of the present study. For all bands defined by these categorical variables, the mean values and standard deviations were calculated for each User Adoption question. Similarly, for the bands defined by the ADL, IADL, and age classifications, the mean age and standard deviation were also calculated.

Furthermore, the dataset was enriched by incorporating two additional composite indices derived from the questionnaire on User Adoption of Assistive Technologies. The Quality Tech Impact Index was calculated as the sum of the scores from questions 1 to 10, which assess technology adoption and perceived impact and ranges from 0 to 30. The Quality of Life Index was calculated as the sum of the recoded scores from questions 11 to 13, which reflect users’ self-perceived quality of life, autonomy, and social relationships and ranges from 0 to 12. For this purpose, questions 11–13, originally rated on a 0–100 scale, were recoded into a 0–4 ordinal scale before computing the composite index. The normality of the data distribution was assessed using the Shapiro–Wilk test. A *p*-value less than 0.05 indicates that the data do not follow a normal distribution [31,32]. Conversely, the homogeneity of variances was assessed through the implementation of Levene’s test [33]. Consequently, given the data did not meet the assumptions of normality and homogeneity of variances, it was decided to adopt non-parametric tests. Among these, the Kruskal–Wallis test [34] was used to compare the medians of the three groups, Low, Medium, and High. The analysis was conducted separately for each independent factor (ADL scale, IADL scale, age classification) whilst maintaining the same dependent variables: the responses to the 13 questions of the User Adoption Questionnaire and the composite scores Quality Tech Impact and Quality of Life Index. When the Kruskal–Wallis test showed significant differences, the Dunn test was used for post hoc analysis. Finally, Spearman correlation analysis was carried out to assess the relationships among age, ADL score, IADL score, Quality Tech Impact, and Quality of Life Index. This test measures the association between two ordinal or non-normally distributed variables using ranks of values. The Spearman correlation coefficient (ρ) ranges from −1, indicating a perfect negative correlation, to +1, indicating a perfect positive correlation, while a value close to 0 indicates no correlation [35]. In the present study, Spearman’s ρ was also interpreted as the effect-size measure for correlation analyses. Ninety-five percent confidence intervals (95% CIs) for Spearman’s ρ were estimated using Fisher’s z transformation, in line with established approaches for interval estimation of rank-based correlation coefficients [36]. *p*-values were corrected using the Bonferroni method [37].

## 3. Results

Table 1 reports the sample size (N), mean, and standard deviation of participants’ age across groups defined by ADL autonomy, IADL instrumental autonomy, and age classification.

Table 2, Table 3 and Table 4 summarises the results of the Kruskal–Wallis tests and the subsequent Dunn’s post hoc analyses for all variables considered. Items 1–13 refer to the USASS questionnaire items; Quality Tech Impact and Quality of Life Index are composite indices derived, respectively, from items 1–10 and items 11–13.

Kruskal–Wallis analyses revealed significant differences among group levels within functional and demographic classifications for several items of the User Adoption Questionnaire. Items assessing technology independence and autonomy-related aspects showed the most consistent effects. Specifically, Question 3 (i.e., Are you independent in using such technology?) and Question 8 (i.e., Will you need help from a family member/caregiver to use assistive technologies?) showed significant differences among Low, Medium, and High groups when ADL, IADL, and age were each considered as independent grouping factors (all *p* < 0.05). Dunn’s post hoc comparisons indicated that participants in the higher ADL and IADL sample-derived groups reported greater independence and a lower need for assistance compared to those in the lower and intermediate groups, while age-based comparisons mainly differentiated younger from older participants.

Several additional items showed significant differences among ADL- and IADL-based groups, indicating that functional autonomy was the most consistent determinant of responses. These items included Question 1 (i.e., Have you ever used technology in the past?), Question 2 (i.e., How much do you enjoy using technology?), Question 5 (i.e., Do you think the use of assistive technologies could improve your quality of life?), Question 6 (i.e., Do you think the use of assistive technologies could increase your autonomy?), Question 7 (i.e., Do you think the use of assistive technologies could improve your self-esteem?), and Question 10 (i.e., How satisfied are you with the way you currently perform this activity?) (all *p* < 0.05 for ADL- and/or IADL-based comparisons). Post hoc analyses consistently showed that the groups with higher sample-derived ADL and IADL levels scored significantly higher than the lower groups and, in some cases, the intermediate groups, whereas Medium–High differences were less systematic. Age-related differences were more limited and less systematic, emerging only for selected items, which suggests that age played a secondary role compared with functional autonomy in these dimensions. Similarly, Questions 11 (i.e., On a scale from 0 to 100, how would you rate your overall current quality of life?), 12 (i.e., On a scale from 0 to 100, how would you rate your autonomy?), and 13 (i.e., On a scale from 0 to 100, how would you rate the quality of your social relationships?), which together contribute to the Quality of Life Index after recoding, showed significant differences among Low, Medium, and High groups within ADL- and IADL-based classifications (all *p* < 0.05). Age-related differences were also observed for some of these items, although the overall pattern remained stronger and more consistent for functional autonomy. Post hoc analyses indicated that participants in the higher ADL and IADL sample-derived groups reported higher scores on these measures compared with the lower groups and, in some cases, the intermediate groups.

No significant differences among Low, Medium, and High groups were observed for Question 4 (Can you rely on help from a family member?) and Question 9 (How important is it for you to be able to perform this activity?), regardless of whether ADL, IADL, or age was used as the grouping factor (*p* > 0.05), indicating homogeneous responses across classifications.

Regarding the composite index, the Quality Tech Impact Index (Item 14), summarising Items 1–10, showed significant differences among ADL- and IADL-based groups (*p* < 0.05). Dunn’s post hoc tests indicated higher scores in the High groups compared to both Low and Medium groups, while no significant differences were detected across age groups. Similarly, the Quality of Life Index (Item 15), derived from recoded Items 11–13, was significantly higher in the High ADL and High IADL groups (*p* < 0.05). A significant age-group effect was also observed, although the overall pattern remained stronger and more consistent for ADL and IADL than for age. Overall, these findings indicate that higher functional autonomy is associated with greater adoption and perceived impact of assistive technologies as well as better self-reported quality of life, whereas age plays a limited role in shaping these outcomes.

**Figure 1:**
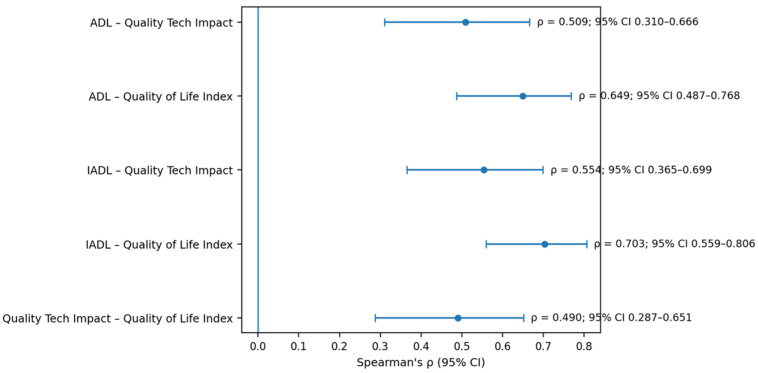
A summary of correlations between functional autonomy and technology-related outcomes. Spearman’s correlation coefficients (ρ) and 95% confidence intervals are shown for the main associations of interest between ADL/IADL and the composite indices Quality Tech Impact and Quality of Life Index as well as for the association between the two composite indices. Positive values indicate that higher functional autonomy or higher technology impact scores are associated with higher scores in the corresponding outcome.

Spearman correlation analyses further showed that ADL and IADL scores were positively associated with both Quality Tech Impact and the Quality of Life Index, with stronger coefficients for functional measures than for age. Because Spearman’s ρ represents the effect-size measure for these associations, the main correlations of interest are reported together with their 95% confidence intervals in Table 5. ADL was positively associated with Quality Tech Impact (ρ = 0.509, 95% CI: 0.310–0.666, *p* = 0.001) and Quality of Life Index (ρ = 0.649, 95% CI: 0.487–0.768, *p* < 0.001). Similarly, IADL was positively associated with Quality Tech Impact (ρ = 0.554, 95% CI: 0.365–0.699, *p* < 0.001) and showed the strongest association with Quality of Life Index (ρ = 0.703, 95% CI: 0.559–0.806, *p* < 0.001). The association between Quality Tech Impact and Quality of Life Index was also positive (ρ = 0.490, 95% CI: 0.287–0.651, *p* < 0.001). After Bonferroni correction, the associations involving ADL/IADL and Quality of Life Index remained significant, as did the association between IADL and Quality Tech Impact. The complete Spearman correlation matrix, unadjusted *p*-values, and Bonferroni-adjusted *p*-values are reported in Supplementary Tables S5–S7, while the main effect sizes and confidence intervals are visually summarised in Figure 1.

Figure 1 provides a graphical summary of the main correlation effect sizes and 95% confidence intervals, highlighting the positive associations between functional autonomy measures and the composite technology-related outcomes.

## 4. Discussion

The analysis of the collected data highlights a significant association between functional autonomy, demographic characteristics, and outcomes related to technology adoption, and quality of life; these findings are consistent with previous works [38,39]. Specifically, differences in technological engagement (Quality Tech Impact), perceived quality of life (Quality of Life Index), and responses to individual items of the User Adoption questionnaire were examined by independently stratifying the sample according to ADL scores, IADL scores, and age.

### 4.1. Adopting Technology: A Reflection of Autonomy

As expected, the results indicate that individuals with higher levels of autonomy (higher ADL and IADL scores) reported greater independence in technology use and a more positive perception of its potential impact on quality of life. This finding confirms a positive association between perceived autonomy and technological adoption. In contrast, this pattern was not observed in the age-based classification, where differences among Low, Medium, and High groups were generally absent. This pattern is in line with the results of the Kruskal–Wallis analyses, which showed that functional autonomy variables (ADL and IADL) were more consistently associated with differences in Question 1 (i.e., Have you ever used technology in the past?) and with the broader pattern of technology-related responses, whereas age-related differences were more limited and less systematic overall. For Question 2 (i.e., How much do you enjoy using technology?), participants in the High group (High ADL/IADL autonomy) expressed higher levels of satisfaction, highlighting functional autonomy as a key factor in positive attitudes toward technology. Although younger participants (Low by age) showed a trend toward greater satisfaction compared to older participants, the lack of statistically significant differences in this clinical sample seems to suggest that age effects are less influential than functional autonomy.

Similarly, responses to Question 3 (i.e., Are you independent in using such technology?) further emphasise that physical and cognitive autonomy contribute to more effective technology use. Furthermore, age also appears to influence perceived technological autonomy, likely due to differences in digital literacy: younger individuals tend to have greater technological experience, whereas older individuals may encounter challenges that reduce their perceived autonomy.

The Quality Tech Impact Index, which summarises the first ten questionnaire items, further confirms that the perceived influence of technology is shaped more by functional limitations than by age. While age affects technological familiarity, functional abilities exert a stronger influence on perceived usefulness and effectiveness. These findings indicate that interventions aimed at improving technological adoption should prioritise the enhancement of functional skills rather than focusing solely on age. Although age remains relevant—particularly with regard to digital literacy—ADL and IADL levels could represent important determinants of successful technology use.

### 4.2. Disparities Between Groups and Clinical Implications

The Quality of Life Index, computed as a composite index of items 11 to 13, reflects patients’ self-reported quality of life-related perceptions rather than the direct impact of technology use. It was significantly higher in the High group for both ADL and IADL classifications; however, the pattern across age-based groups was less consistent and less pronounced than that observed for ADL and IADL classifications. Several factors may contribute to this discrepancy. First, cognitive or physical barriers may limit the ability of individuals with low autonomy to use technological aids effectively, thereby reducing their perceived benefit. Second, family support may mediate the impact of technology, as suggested by the relationship between IADL scores and participants’ perceptions of social support, assessed through Questions 4 (Can you rely on help from a family member?) and 8 (Will you need help from a family member/caregiver to use assistive technologies?).

From a clinical perspective, these findings highlight the need for targeted interventions aimed at strengthening both functional capacities and digital self-efficacy. Training programs and psychological support may enhance confidence and promote greater acceptance of assistive technologies [40]; in addition, these activities might be used as a strong predictor of patients’ behavioural intentions [41].

### 4.3. Correlation Analysis

Correlation analyses provided further support for the association between functional autonomy and technology-related outcomes. When interpreted as effect-size measures, Spearman’s ρ values indicated moderate-to-strong positive associations between functional autonomy and the two composite outcomes. In particular, IADL showed the strongest association with the Quality of Life Index (ρ = 0.703, 95% CI: 0.559–0.806, *p* < 0.001), suggesting that instrumental autonomy may be especially relevant for perceived well-being in the transition from inpatient rehabilitation to home-based technology-supported care. Age showed weaker negative correlations with both composite indices in the unadjusted analysis, whereas the most robust associations after Bonferroni correction remained those involving ADL and IADL. These findings reinforce the notion that functional abilities, rather than chronological age, are significant drivers of successful technology adoption and its perceived benefits in this population. The findings emphasise the importance of employing clinical scales that are specifically designed to assess an individual’s capacity to perform daily activities in conducting studies that aim to evaluate the level of readiness for technology utilisation.

### 4.4. Comparison with Previous Literature

As outlined in the introduction, the existing literature on technology adoption has predominantly centred on community-dwelling older adults or general smart-home contexts [15,17]. Consequently, the precise dynamics of technology adoption during the transition from inpatient neuromotor rehabilitation to home-based care have remained a significant research gap [17]. Our findings directly address this gap. While chronological age is frequently analysed in broader aging-in-place studies [15], our analysis reveals that within a rehabilitation setting framework, functional autonomy is a far more consistent determinant of a patient’s readiness to adopt assistive technologies. This insight expands upon existing digital maturity models [21], suggesting that for vulnerable clinical populations transitioning home, targeted assessments of functional capacity are critical to inform the design of effective technological solutions. Building on this, our findings highlight ADL and IADL as a central determinant of quality of life and a key factor influencing the uptake of assistive technologies among people with disabilities. Consistent with prior evidence, higher functional autonomy is generally associated with better health-related outcomes and greater user satisfaction [42,43,44]. The effectiveness of assistive technologies depends not only on their technical performance but also on their accessibility, adaptability, and capacity to accommodate users’ changing needs. These aspects are particularly salient for individuals with reduced autonomy or heightened frailty [42,45,46]. In this regard, the questionnaire-based assessment adopted in the study supports the value of early and sustained user involvement. Specifically, participatory and co-design approaches may facilitate adoption by improving perceived relevance, usability, and the fit between device functionalities and everyday contexts of use [47,48]. An additional finding concerns the role of age. Although chronological age is often treated as a barrier to adoption of technology, the relationship appears complex and non-linear. The pattern observed in this study is therefore not necessarily contradictory to the broader literature, as age-related differences in technology use are frequently mediated by variables such as cognitive functioning, self-efficacy, prior experience, perceived usefulness, motivation, digital literacy, social support, and confidence rather than by age per se. When these mediators are accounted for, the direct effect of chronological age on adoption often attenuates or becomes non-significant [48,49]. In the present work, these mediating factors could not be examined systematically due to limited availability of contextual information. Overall, functional autonomy emerges as a pivotal factor shaping not only the likelihood of adoption but also the effectiveness and design requirements of assistive technologies. Both clinical interventions and design strategies should consider heterogeneity in autonomy levels (alongside chronological age) to maximise benefits across user groups. Furthermore, as noted in the introduction, the adoption of a structured questionnaire contributes to addressing shortcomings in the systematisation and reporting of user-centred design practices, thereby enforcing the need for stronger implementation standards and more evidence on long-term outcomes. Finally, structured training initiatives and ongoing support systems are likely to be particularly beneficial for users with lower autonomy, as they can enhance confidence and technological competence and promote sustained use over time.

### 4.5. Limitations

This study has several limitations. The relatively small, single-centre sample limits generalisability, and participants were primarily in intensive neuromotor rehabilitation, potentially biasing results. The cross-sectional design prevents causal inference between functional autonomy and technology adoption. Outcomes were based on self-report, collected with an ad hoc non-validated tool, and cognitive status and digital literacy were partially assessed, which may influence responses. In addition, the study addressed assistive technologies as a broad category of potential home-based solutions and did not distinguish between specific device types or technology domains; therefore, the findings should be interpreted as referring to general attitudes and expectations toward assistive technology rather than to acceptance of any single product or platform. Moreover, ADL, IADL, and age were categorised using sample-derived distribution thresholds to enable exploratory group comparisons; these categories were not intended as clinical levels and may have reduced the information carried by the original continuous scores. Therefore, findings based on these categories should be interpreted as exploratory comparisons across sample-derived analytical strata rather than as differences between clinically meaningful patient subgroups. Moreover, groups the stratification used did not reflect direct clinical differences but were based on statistical and methodological choice. Finally, no longitudinal follow-up was available to confirm sustained technology use after discharge. Despite these limitations, the study provides a first attempt to explore a still under-investigated topic for which, to the best of our knowledge, no standardised tools currently exist to effectively assess this area.

## 5. Conclusions

This study highlights functional autonomy, particularly as measured by ADL and IADL as the primary determinant of both technology adoption and perceived quality of life in patients transitioning from inpatient neuromotor rehabilitation to home-based care. Unlike chronological age, which showed limited and inconsistent influence, functional capacity emerged as a more reliable predictor of engagement with assistive technologies, perceived usefulness, and overall well-being. These findings contribute to the existing body of knowledge by emphasising the need to move beyond age-based assumptions and instead adopt a more nuanced, function-centred approach when designing, prescribing, and implementing technological solutions in clinical populations. From a practical perspective, the results support the development of tailored interventions that integrate functional training, digital literacy enhancement, and user-centred design strategies to improve adoption and sustained use. Future research should address the limitations of this study by including larger and more diverse samples, employing longitudinal designs to assess long-term outcomes, and incorporating more comprehensive measures of cognitive status, digital competence, and psychosocial factors. Additionally, further validation of standardised tools to assess technology readiness in clinical populations will be essential to guide both clinical decision-making and the development of effective assistive technologies.

## Figures and Tables

**Table 1:** Sample size (N), mean, and standard deviation of participants’ age according to ADL autonomy (Low, Medium, High), IADL instrumental autonomy (Low, Medium, High), and age classification (Low, Medium, High).

	ADL Scale		IADL Scale		Age	
Classes	N	Mean ± Std	N	Mean ± Std	N	Mean ± Std
Low (autonomy/instrumental autonomy/age)	18	53.54 ± 16.69	24	50.39 ± 15.83	20	39.05 ± 9.40
Medium (autonomy/instrumental autonomy/age)	23	62.17 ± 18.05	12	64.83 ± 14.08	17	55.76 ± 3.11
High (autonomy/instrumental autonomy/age)	28	64.17 ± 10.92	33	68.46 ± 11.55	32	73.59 ± 6.91

**Table 2:** Kruskal–Wallis and Dunn’s post hoc results for user adoption, technology impact, and quality of life by ADL (ns refers to not significant). Question 14 refers to Quality Tech Impact, and question 15 refers to Quality of Life Index.

ADL Scales				
Questions	Mean ± Std	Low vs. Medium	Low vs. Medium	Medium vs. High
1	2.39 ± 0.92			
	1.50 ± 0.79	ns	0.005	ns
	1.96 ± 1.07			
2	1.79 ± 1.29			
	0.50 ± 0.79	ns	0.005	ns
	1.87 ± 1.14			
3	2.32 ± 0.86			
	1.06 ± 0.73	0.001	0.000	ns
	2.13 ± 0.69			
4	2.04 ± 1.00			
	1.50 ± 0.99	ns	ns	ns
	2.27 ± 0.77			
5	2.46 ± 0.69			
	1.67 ± 0.77	0.046	ns	ns
	2.48 ± 0.60			
6	2.57 ± 0.69			
	1.72 ± 0.89	0.008	0.004	ns
	2.35 ± 0.57			
7	2.43 ± 0.69			
	1.56 ± 0.86	ns	0.001	ns
	2.23 ± 0.53			
8	0.93 ± 0.98			
	1.83 ± 0.99	ns	0.001	ns
	1.00 ± 0.93			
9	2.64 ± 0.58			
	2.00 ± 1.07	ns	ns	ns
	1.95 ± 1.22			
10	1.82 ± 0.96			
	0.81 ± 1.28	0.041	0.013	ns
	0.55 ± 1.00			
11	64.6 ± 21.6			
	30.7 ± 24.3	ns	0.040	0.006
	49.2 ± 20.9			
12	71.7 ± 22.0			
	25.0 ± 27.9	ns	0.000	ns
	46.8 ± 18.8			
13	80.1 ± 18.0			
	43.8 ± 32.2	ns	0.000	0.013
	66.8 ± 21.2			
14	20.4 ± 4.95			
	13.7 ± 5.29	0.043	0.000	ns
	17.9 ± 3.76			
15	6.61 ± 2.22			
	2.39 ± 2.09	ns	0.000	0.0045
	4.17 ± 1.67			

**Table 3:** Kruskal–Wallis and Dunn’s post hoc results for user adoption, technology impact, and quality of life by IADL (ns refers to not significant). Question 14 refers to Quality Tech Impact, and question 15 refers to Quality of Life Index.

IADL Scales				
Questions	Mean ± Std	Low vs. Medium	Low vs. Medium	Medium vs. High
1	2.48 ± 0.76			
	1.50 ± 0.88	ns	0.000	ns
	1.75 ± 1.22			
2	1.88 ± 1.22			
	0.92 ± 1.18	ns	0.008	ns
	1.50 ± 1.17			
3	2.42 ± 0.71			
	1.13 ± 0.80	0.007	0.000	ns
	2.17 ± 0.58			
4	2.06 ± 0.97			
	1.75 ± 0.94	ns	ns	ns
	2.18 ± 0.98			
5	2.52 ± 0.68			
	1.88 ± 0.80	ns	0.001	ns
	2.33 ± 0.65			
6	2.52 ± 0.67			
	1.83 ± 0.82	ns	0.039	ns
	2.50 ± 0.67			
7	2.39 ± 0.66			
	1.74 ± 0.86	ns	0.010	ns
	2.17 ± 0.58			
8	0.78 ± 0.87			
	2.04 ± 0.86	0.000	0.000	ns
	0.58 ± 0.51			
9	2.38 ± 1.02			
	2.00 ± 1.10	ns	ns	ns
	2.33 ± 0.71			
10	1.58 ± 1.06			
	0.68 ± 1.13	ns	0.044	ns
	0.80 ± 1.32			
11	65.0 ± 19.6			
	35.8 ± 23.1	ns	0.000	0.041
	42.5 ± 26.3			
12	71.6 ± 21.4			
	30.0 ± 25.2	ns	0.000	0.010
	40.0 ± 20.0			
13	80.4 ± 20.2			
	48.8 ± 29.2	ns	0.000	ns
	63.3 ± 20.2			
14	20.0 ± 5.09			
	15.1 ± 5.14	ns	0.002	ns
	17.4 ± 3.92			
15	6.39 ± 2.30			
	2.88 ± 1.96	ns	ns	0.006
	3.67 ± 1.56			

**Table 4:** Kruskal–Wallis and Dunn’s post hoc results for user adoption, technology impact, and quality of life by age groups (ns refers to not significant). Question 14 refers to Quality Tech Impact, and question 15 refers to Quality of Life Index.

Age				
Questions	Mean ± Std	Low vs. Medium	Low vs. Medium	Medium vs. High
1	1.59 ± 0.98			
	2.50 ± 0.76	ns	ns	ns
	2.24 ± 0.97			
2	1.22 ± 1.21			
	1.90 ± 1.17	ns	ns	ns
	1.47 ± 1.37			
3	1.53 ± 0.95			
	2.50 ± 0.69	ns	ns	ns
	2.00 ± 0.79			
4	2.06 ± 0.81			
	1.75 ± 0.89	ns	ns	ns
	2.18 ± 1.18			
5	2.13 ± 0.75			
	2.50 ± 0.71	ns	ns	ns
	2.24 ± 0.83			
6	2.19 ± 0.69			
	2.40 ± 0.82	ns	ns	ns
	2.29 ± 0.92			
7	1.94 ± 0.73			
	2.20 ± 0.77	ns	ns	ns
	2.41 ± 0.80			
8	1.78 ± 0.87			
	0.70 ± 0.86	ns	0.001	0.001
	0.63 ± 0.89			
9	2.33 ± 1.02			
	2.32 ± 1.10	ns	ns	ns
	1.80 ± 0.71			
10	1.14 ± 1.33			
	1.25 ± 1.12	ns	ns	ns
	0.70 ± 0.95			
11	43.2 ± 26.47			
	63.2 ± 23.52	ns	0.020	ns
	50.0 ± 22.21			
12	41.3 ± 32.1			
	66.0 ± 24.8	ns	0.009	ns
	53.1 ± 22.4			
13	58.7 ± 29.0			
	80.7 ± 23.1	0.047	0.011	ns
	63.1 ± 21.8			
14	17.3 ± 5.22			
	20.1 ± 5.35	ns	ns	ns
	16.2 ± 4.99			
15	3.97 ± 2.40			
	6.30 ± 2.36	0.034	0.010	ns
	4.18 ± 2.67			

**Table 5:** Spearman correlation effect sizes and 95% confidence intervals for the main associations of interest. Spearman’s ρ was interpreted as the effect-size measure. CI = confidence interval.

Association	Spearman’s ρ	95% CI	*p*-Value	Bonferroni-Adjusted *p*
ADL—Quality Tech Impact	0.509	0.310–0.666	0.001	ns
ADL—Quality of Life Index	0.649	0.487–0.768	<0.001	<0.001
IADL—Quality Tech Impact	0.554	0.365–0.699	<0.001	0.010
IADL—Quality of Life Index	0.703	0.559–0.806	<0.001	<0.001
Quality Tech Impact—Quality of Life Index	0.490	0.287–0.651	<0.001	ns

## Data Availability

The data are available upon request from the corresponding author. The data are not publicly available due to privacy and ethical restrictions, as they contain sensitive patient information that could potentially compromise the anonymity of the participants, in accordance with the informed consent processed at the time of the study.

## Supplementary Materials

The following supporting information can be downloaded at https://www.mdpi.com/article/10.3390/healthcare14101355/s1, Table S1: Descriptive statistics of user adoption, technology impact, and quality of life; Table S2: ADL classification groups based on sample-derived distribution thresholds; Table S3: IADL classification groups based on sample-derived distribution thresholds; Table S4: Age classification groups based on sample-derived distribution thresholds; Table S5: Spearman correlation matrix for the selected variables of interest; Table S6: *p*-values associated with Spearman correlation coefficients; Table S7: Bonferroni-adjusted *p*-values for Spearman correlations. The complete USASS questionnaire is also provided, including the Activities of Daily Living (ADL) assessment form and the Instrumental Activities of Daily Living (IADL) assessment form.

## Abbreviations

The following abbreviations are used in this manuscript:

SAALUS	Smart Ambient Assisted Living Ubiquitous System
ADL	Activity Daily Living
IADL	Instrumental Activity Daily Living
USASS	Usability Assessment Survey
