# Supplementary material for: Exploring the Needs and Expectations of Inpatients Towards Assistive Technologies During Neuromotor Rehabilitation
Source: Healthcare (Basel). 2026 May 15;14(10):1355. doi: 10.3390/healthcare14101355 (PMC13205515; doi:10.3390/healthcare14101355)
Supplement: Supplementary file 1 [file healthcare-14-01355-s001.zip › healthcare-4232927-supplementary.pdf]

1. Supplementary Materials

The purpose of this chapter is to provide and describe the supplementary materials accompanying the study entitled "Exploring the Needs and Expectations of In-Patients Towards Assistive Technologies During Neuromotor Rehabilitation.". These materials include additional data, methodological details that complement the main body of the research.

Descriptive Statistics and Sample-Derived Distribution Thresholds

Table S1 presents the descriptive statistics (mean ± standard deviation) for the USASS questionnaire and for the two composite indices included in the analysis. Specifically, Items 1–13 correspond to the original USASS questionnaire items. In addition, Item 14 refers to the Quality Tech Impact index, computed as the sum of USASS items 1–10, with a possible score range from 0 to 30. Item 15 refers to the Quality of Life Index, computed as the sum of the recoded scores of USASS items 11–13, with a possible score range from 0 to 12. For the calculation of this latter index, items 11–13, originally recorded on a 0–100 scale, were recoded into a 0–4 ordinal scale before summation. Therefore, in Table S1, Items 14 and 15 should be interpreted as derived composite indices rather than as additional questionnaire items.

Table S2, Table S3 and Table S4 present the distribution of participants across the three sample-derived ordered groups (low, medium, and high) identified for ADL classification, IADL classification, and age. The grouping was performed using sample-derived distribution thresholds to create three ordered categories (≤Q1, Q1–Q2, >Q2) for exploratory analyses. These groups do not correspond to four quartiles of equal size and should not be interpreted as clinical levels. Each table reports the minimum and maximum observed values defining each group range, together with the number of participants included in that category. For each group, the count of participants is shown, classified according to two thresholds: the minimum (Min) score representing the lowest value of ADL, IADL, or Age within that range, and the maximum (Max) score indicating the highest value for the same variable in that range.

Table S1. Descriptive statistics of user adoption, technology impact and quality of life.

		Mean ± Std		
Questions	Classes	ADL Scales	I.A.D.L. Scales	Age
1	high (autonomy/ instrumental autonomy/ age)	2,39 ± 0,92	2,48 ± 0,76	1,59 ± 0,98
	medium (autonomy/ instrumental autonomy/ age)	1,50 ± 0,79	1,50 ± 0,88	2,50 ± 0,76
	low (autonomy/ instrumental autonomy/ age)	1,96 ± 1,07	1,75 ± 1,22	2,24 ± 0,97
2	high (autonomy/ instrumental autonomy/ age)	1,79 ± 1,29	1,88 ± 1,22	1,22 ± 1,21
	medium (autonomy/ instrumental autonomy/ age)	0,50 ± 0,79	0,92 ± 1,18	1,90 ± 1,17
	low (autonomy/ instrumental autonomy/ age)	1,87 ± 1,14	1,50 ± 1,17	1,47 ± 1,37
3	high (autonomy/ instrumental autonomy/ age)	2,32 ± 0,86	2,42 ± 0,71	1,53 ± 0,95
	medium (autonomy/ instrumental autonomy/ age)	1,06 ± 0,73	1,13 ± 0,80	2,50 ± 0,69
	low (autonomy/ instrumental autonomy/ age)	2,13 ± 0,69	2,17 ± 0,58	2,00± 0,79
4	high (autonomy/ instrumental autonomy/ age)	2,04 ± 1,00	2,06 ± 0,97	2,06 ± 0,81
	medium (autonomy/ instrumental autonomy/ age)	1,50 ± 0,99	1,75 ± 0,94	1,75 ± 0,89
	low (autonomy/ instrumental autonomy/ age)	2,27 ± 0,77	2,18 ± 0,98	2,18 ± 1,18
5	high (autonomy/ instrumental autonomy/ age)	2,46 ± 0,69	2,52 ± 0,68	2,13 ± 0,75
	medium (autonomy/ instrumental autonomy/ age)	1,67 ± 0,77	1,88 ± 0,80	2,50 ± 0,71
	low (autonomy/ instrumental autonomy/ age)	2,48 ± 0,60	2,33 ± 0,65	2,24 ± 0,83
6	high (autonomy/ instrumental autonomy/ age)	2,57 ± 0,69	2,52 ± 0,67	2,19 ± 0,69
	medium (autonomy/ instrumental autonomy/ age)	1,72 ± 0,89	1,83 ± 0,82	2,40 ± 0,82
	low (autonomy/ instrumental autonomy/ age)	2,35 ± 0,57	2,50 ± 0,67	2,29 ± 0,92

7	high (autonomy/ instrumental autonomy/ age)	2,43 ± 0,69	2,39 ± 0,66	1,94 ± 0,73
	medium (autonomy/ instrumental autonomy/ age)	1,56 ± 0,86	1,74 ± 0,86	2,20 ± 0,77
	low (autonomy/ instrumental autonomy/ age)	2,23 ± 0,53	2,17 ± 0,58	2,41 ± 0,80
8	high (autonomy/ instrumental autonomy/ age)	0,93 ± 0,98	0,78 ± 0,87	1,78 ± 0,87
	medium (autonomy/ instrumental autonomy/ age)	1,83 ± 0,99	2,04 ± 0,86	0,70 ± 0,86
	low (autonomy/ instrumental autonomy/ age)	1,00 ± 0,93	0,58 ± 0,51	0,63 ± 0,89
9	high (autonomy/ instrumental autonomy/ age)	2,64 ± 0,58	2,38 ± 1,02	2,33 ± 1,02
	medium (autonomy/ instrumental autonomy/ age)	2,00 ± 1,07	2,00 ± 1,10	2,32 ± 1,10
	low (autonomy/ instrumental autonomy/ age)	1,95 ± 1,22	2,33 ± 0,71	1,80 ± 0,71
10	high (autonomy/ instrumental autonomy/ age)	1,82 ± 0,96	1,58 ± 1,06	1,14 ± 1,33
	medium (autonomy/ instrumental autonomy/ age)	0,81 ± 1,28	0,68 ± 1,13	1,25 ± 1,12
	low (autonomy/ instrumental autonomy/ age)	0,55 ± 1,00	0,80 ± 1,32	0,70 ± 0,95
11	high (autonomy/ instrumental autonomy/ age)	64,6 ± 21,6	65,0 ± 19,6	43,2 ±26,47
	medium (autonomy/ instrumental autonomy/ age)	30,7 ± 24,3	35,8 ± 23,1	63,2 ± 23,52
	low (autonomy/ instrumental autonomy/ age)	49,2 ± 20,9	42,5 ± 26,3	50,0 ± 22,21
12	high (autonomy/ instrumental autonomy/ age)	71,7 ± 22,0	71,6 ± 21,4	41,3 ± 32,1
	medium (autonomy/ instrumental autonomy/ age)	25,0 ± 27,9	30,0 ± 25,2	66,0 ± 24,8
	low (autonomy/ instrumental autonomy/ age)	46,8 ± 18,8	40,0 ± 20,0	53,1 ± 22,4
13	high (autonomy/ instrumental autonomy/ age)	80,1 ± 18,0	80,4 ± 20,2	58,7 ± 29,0
	medium (autonomy/ instrumental autonomy/ age)	43,8 ± 32,2	48,8 ± 29,2	80,7 ± 23,1
	low (autonomy/ instrumental autonomy/ age)	66,8 ± 21,2	63,3 ± 20,2	63,1 ± 21,8
14	high (autonomy/ instrumental autonomy/ age)	20,4 ± 4,95	20,0 ± 5,09	17,3 ± 5,22
	medium (autonomy/ instrumental autonomy/ age)	13,7 ± 5,29	15,1 ± 5,14	20,1 ± 5,35
	low (autonomy/ instrumental autonomy/ age)	17,9 ± 3,76	17,4 ± 3,92	16,2 ± 4,99
15	high (autonomy/ instrumental autonomy/ age)	6,61 ± 2,22	6,39 ± 2,30	3,97 ± 2,40
	medium (autonomy/ instrumental autonomy/ age)	2,39 ± 2,09	2,88 ± 1,96	6,30 ± 2,36
	low (autonomy/ instrumental autonomy/ age)	4,17 ± 1,67	3,67 ± 1,56	4,18 ± 2,67

Table S2. ADL classification groups based on sample-derived distribution thresholds.

ADL Score Range	Min	Max	Count
high autonomy	6	6	28
medium autonomy	2	5	23
low autonomy	0	1	18

Table S3. IADL classification groups based on sample-derived distribution thresholds.

IADL Score Range	Min	Max	Count
high instrumental autonomy	5	8	33
medium instrumental autonomy	4	4	12
low instrumental autonomy	1	3	24

Table S4. Age classification groups based on sample-derived distribution thresholds.

Age Score Range	Min	Max	Count
high age	61	87	32
medium age	50	60	17
low age	23	49	20

Spearman's correlation results tables

Table S5 presents the Spearman correlation matrix for the selected variables of interest. This analysis was conducted to explore the strength and direction of monotonic relationships among the study variables, providing insight into potential associations that may influence user adoption, perceived technology impact, and quality of life outcomes.

Table S6 reports the p-values associated with the Spearman correlation coefficient and Table S7 presents the p-values adjusted using the Bonferroni correction, applied to control for the increased risk of Type I error due to multiple comparisons.

Table S5. Spearman correlation matrix for the selected variables of interest.

	**Age**	**A.D.L Score**	**I.A.D.L. Score**	**Quality Tech Impact**	**Quality of Life Index**
**Age**	1,000	-0,468	-0,460	-0,291	-0,357
**A.D.L Score**	-0,468	1,000	0,888	0,509	0,649
**I.A.D.L. Score**	-0,460	0,888	1,000	0,554	0,703
**Quality Tech Impact**	-0,291	0.509	0,554	1,00	0,490
**Quality of Life Index**	-0,357	0,649	0,703	0,490	1,00

Table S6. P-values associated with Spearman correlation coefficients.

	**Age**	**A.D.L Score**	**I.A.D.L. Score**	**Quality Tech Impact**	**Quality of Life Index**
**Age**	ns	**0,001**	**0,001**	0,040	0,010

**A.D.L Score**	0,001	ns	**<0,001**	0,001	**<0,001**
**I.A.D.L. Score**	0,001	**<0,001**	ns	**<0,001**	**<0,001**
**Quality Tech Impact**	0,040	**0,001**	<0.001	**ns**	**<0,001**
**Quality of Life Index**	0,010	**<0,001**	**<0,001**	**<0,001**	**ns**

Table S7. Bonferroni adjusted p-values for Spearman correlations

	**Age**	**A.D.L Score**	**I.A.D.L. Score**	**Quality Tech Impact**	**Quality of Life Index**
**Age**	ns	ns	ns	ns	ns
**A.D.L Score**	ns	ns	**0,000**	ns	**0,000**
**I.A.D.L. Score**	ns	**0,000**	ns	**0,010**	**0,000**
**Quality Tech Impact**	ns	**ns**	0,000	**ns**	**ns**
**Quality of Life Index**	ns	**0,000**	0,000	**ns**	**ns**

2. Questionnaire

Activitis of Daily Living (ADL) Assessment Form

A. Bathing (tub, shower, sponge bath)

1. Bathes self completely (enters and exits the tub or shower independently). – (**1**)
2. Requires assistance only for washing one part of the body (e.g., back). – (**1**)
3. Requires assistance for more than one part of the body. – (**0**)

B. Dressing (retrieving clothes from closet/drawers, including underwear, fastening or using suspenders if needed)

1. Retrieves clothing and dresses completely without assistance. – (**1**)
2. Dresses independently except for tying shoes. – (**1**)
3. Needs assistance in obtaining clothing or dressing, or remains partially/completely undressed. – (**0**)

C. Toilet Use (going to the toilet for urination and defecation, cleaning, dressing afterward)

1. Goes to the toilet, cleans, and dresses without assistance (may use aids such as cane, walker, wheelchair, bedpan, or commode, and empties them in the morning). – (**1**)
2. Requires assistance in going to the toilet, cleaning, dressing, or using/emptying the bedpan or commode. – (**0**)
3. Does not go to the toilet for elimination. – (**0**)

D. Transfers

1. Moves in and out of bed and chair independently (may use canes or walker). – (**1**)
2. Performs transfers with assistance. – (**0**)
3. Confined to bed, does not get up. – (**0**)

E. Bowel and Bladder Control

1. Complete control over bowel and bladder. – (**1**)
2. Occasional "accidents." – (**0**)
3. Requires supervision, uses catheter, or is incontinent. – (**0**)

F. Feeding

1. Eats without assistance. – (**1**)
2. Requires help only for cutting meat or buttering bread. – (**1**)
3. Requires assistance to bring food to mouth or is partially/totally fed parenterally. – (**0**)

Instrumental Activities of Daily Living (IADL) Assessment Form

A. Ability to Use the Telephone

1. Uses the telephone on own initiative: looks up numbers and dials them. – **(1)**
2. Dials only a few well-known numbers. – **(1)**
3. Answers the phone but does not dial numbers. – **(1)**
4. Unable to use the telephone. – **(0)**

B. Shopping

1. Takes care of all shopping needs independently. – **(1)**
2. Capable of small purchases only. – **(0)**
3. Requires accompaniment for any kind of shopping. – **(0)**
4. Completely unable to do any shopping. – **(0)**

C. Food Preparation

1. Plans, prepares, and serves meals independently. – **(1)**
2. Prepares meals only if all ingredients are supplied. – **(0)**
3. Can only reheat prepared food or inconsistently prepares meals, resulting in poor diet. – **(0)**
4. Needs prepared meals and must be served. – **(0)**

D. Housekeeping

1. Maintains house independently or with occasional help for heavy work. – **(1)**
2. Performs light daily tasks such as dishwashing or bed making. – **(1)**
3. Performs light tasks but fails to maintain adequate cleanliness. – **(1)**
4. Requires assistance for all household cleaning. – **(0)**
5. Completely disinterested in any household tasks. – **(0)**

E. Laundry

1. Does personal laundry completely. – **(1)**
2. Washes small items only. – **(1)**
3. All laundry must be done by others. – **(0)**

F. Mode of Transportation

1. Travels independently using public transportation or drives own car. – **(1)**
2. Uses taxi but cannot manage public transport. – **(1)**
3. Travels by public transport only when assisted or accompanied. – **(1)**
4. Travels by car or taxi only when assisted by others. – **(0)**
5. Does not travel at all. – **(0)**

G. Responsibility for Own Medications

1. Takes medication correctly on own. – **(1)**
2. Takes medication if prepared and set out in advance. – **(0)**
3. Unable to take medication independently. – **(0)**

H. Ability to Handle Finances

1. Manages all financial matters independently (bills, checks, rent, bank transactions). – (**1)**
2. Manages daily expenses and minor transactions but needs help for major ones (bank, large purchases, etc.). – (**1)**
3. Unable to handle money properly. – (**0)**

USASS

1. Have you ever used technology in the past?

1. Always – (**3**)
2. Often – (**2**)
3. Sometimes – (**1**)
4. Never – (**0**)

2. How much do you enjoy using technology?

1. Very much – (**3**)
2. Quite a lot – (**2**)
3. A little – (**1**)
4. Not at all – (**0**)

3. Are you independent in using such technology?

1. Always – (**3**)
2. Often – (**2**)
3. Sometimes – (**1**)
4. Never – (**0**)

4. Can you rely on help from a family member?

1. Always – (**3**)
2. Often – (**2**)
3. Sometimes – (**1**)
4. Never – (**0**)

5. Do you think the use of assistive technologies could improve your quality of life?

1. Always – (**3**)
2. Often – (**2**)
3. Sometimes – (**1**)
4. Never – (**0**)

6. Do you think the use of assistive technologies could increase your autonomy?

1. Always – (**3**)
2. Often – (**2**)
3. Sometimes – (**1**)
4. Never – (**0**)

7. Do you think the use of assistive technologies could improve your self-esteem?

1. Always – (**3**)
2. Often – (**2**)
3. Sometimes – (**1**)
4. Never – (**0**)

8. Will you need help from a family member/caregiver to use assistive technologies?

1. Never – (**3**)
2. Sometimes– (**2**)
3. Often– (**1**)
4. Always – (**0**)

9. How important is it for you to be able to perform this activity?

1. Extremely important – (**3**)
2. Very important – (**2**)
3. Fairly important – (**1**)
4. Slightly important – (**0**)

10. How satisfied are you with the way you currently perform this activity?

1. Extremely satisfied – (**3**)
2. Very satisfied – (**2**)
3. Fairly satisfied – (**1**)
4. Slightly satisfied – (**0**)

11. On a scale from 0 to 100, how would you rate your overall current quality of life?
_____/100
12. On a scale from 0 to 100, how would you rate your autonomy?
_____/100
13. On a scale from 0 to 100, how would you rate the quality of your social relationships?
_____/100
